# Forecasting deforestation and carbon emissions in tropical developing countries facing demographic expansion: a case study in Madagascar

**DOI:** 10.1002/ece3.550

**Published:** 2013-05-03

**Authors:** Ghislain Vieilledent, Clovis Grinand, Romuald Vaudry

**Affiliations:** 1 Cirad – UPR BSEFF34398 Montpellier, Cedex 5, France; 2 Cirad-Madagascar – DP Forêt et BiodiversitéBP 853, Ambatobe, 101-Antananarivo, Madagascar; 3 GoodPlanet – Fondation GoodPlanetDomaine de Longchamp, 1 carrefour de Longchamp, F-75116, Paris, France

**Keywords:** Anthropogenic deforestation, biodiversity conservation, climate change, GRASS GIS, greenhouse gas emission, land use change, logistic regression model, phcfM R package, population growth, REDD+

## Abstract

Anthropogenic deforestation in tropical countries is responsible for a significant part of global carbon dioxide emissions in the atmosphere. To plan efficient climate change mitigation programs (such as REDD+, Reducing Emissions from Deforestation and forest Degradation), reliable forecasts of deforestation and carbon dioxide emissions are necessary. Although population density has been recognized as a key factor in tropical deforestation, current methods of prediction do not allow the population explosion that is occurring in many tropical developing countries to be taken into account. Here, we propose an innovative approach using novel computational and statistical tools, including R/GRASS scripts and the new phcfM R package, to model the intensity and location of deforestation including the effect of population density. We used the model to forecast anthropogenic deforestation and carbon dioxide emissions in five large study areas in the humid and spiny-dry forests of Madagascar. Using our approach, we were able to demonstrate that the current rapid population growth in Madagascar (+3.39% per year) will significantly increase the intensity of deforestation by 2030 (up to +1.17% per year in densely populated areas). We estimated the carbon dioxide emissions associated with the loss of aboveground biomass to be of 2.24 and 0.26 tons per hectare and per year in the humid and spiny-dry forest, respectively. Our models showed better predictive ability than previous deforestation models (the figure of merit ranged from 10 to 23). We recommend this approach to reduce the uncertainty associated with deforestation forecasts. We also underline the risk of an increase in the speed of deforestation in the short term in tropical developing countries undergoing rapid population expansion.

## Introduction

Tropical forests provide various ecosystem services both at the global and local scale (Kremen and Ostfeld 2005). They contain more species than any other ecosystem on emerged lands (Gibson et al. 2011) and are large carbon sinks (Pan et al. 2011). Locally, tropical forests have the capacity to regulate water supply and to provide high-quality water to surrounding populations (Bradshaw et al. 2007). Thus, tropical deforestation is responsible not only for a major decline in biodiversity (Gibson et al. 2011), but also for a considerable proportion (6–17%) of global carbon dioxide emissions that affect climate change (IPCC 2007; Baccini et al. 2012) and is the first step toward land desertification (Geist 2005; Xu et al. 2011). Around 13 million hectares of tropical forest are deforested each year around the world (FAO 2005). Within the climate change mitigation framework, accurate forecasts of deforestation and carbon dioxide emissions are essential for the application of the REDD+ Programme, which aims at “Reducing Emissions from Deforestation and forest Degradation” (Olander et al. 2008). The ability to forecast deforestation and carbon emissions is determined by the availability of reliable data sets, together with progress in methodology, computation, and statistics (Clark et al. 2001).

Population density is recognized as one of the main factors that determine deforestation intensity in the tropics (López-Carr 2004; López-Carr et al. 2005). An increase in population density leads to stronger pressure on forests due to harvesting of wood for construction or fuel, or through slash-and-burn for cattle grazing and agriculture (Allen and Barnes 1985; Kaimowitz and Angelsen 1998; Geist and Lambin 2001). Additionally, in many tropical developing countries, especially in Africa, the demographic transition is not over (the demographic transition refers to the transition from high birth and death rates to low birth and death rates as a country develops from a preindustrial to an industrialized economic system; Kingsley 1945). In these countries, death rates have been decreasing but birth rates remain high. The inevitable outcome is a population expansion characterized by a high growth rate and a short doubling-time (amount of time needed for a given population to double) (United Nations 2011; Raftery et al. 2012). Several authors have already tried to statistically estimate the relationship between population density and deforestation intensity (Allen and Barnes 1985; Apan and Peterson 1998; Pahari and Murai 1999; Agarwal et al. 2005; López-Carr et al. 2008; Gorenflo et al. 2011). Most studies identified an increase in deforestation intensity with an increase in population density but in several cases, the effect was weak (Agarwal et al. 2005) or not statistically significant (Apan and Peterson 1998; Gorenflo et al. 2011). Apart from the fact that many political, socioeconomic and ecological factors that are different from population density might explain deforestation intensity (Geist and Lambin 2001), several methodological problems arise when trying to estimate the effect of population density on deforestation intensity.

A common pitfall of deforestation models is using spatial explanatory factors such as distance to forest edge (Gorenflo et al. 2011), or elevation (Apan and Peterson 1998; Agarwal et al. 2005) in association with population density to predict the intensity of deforestation. The effects of such spatial factors are usually highly significant and of high magnitude compared to population density for which available data are usually at a much coarser resolution (Agarwal et al. 2005). Elevation and distance to forest edge, which are proxies for the accessibility of the forest, are usually strongly negatively correlated with the probability of deforestation (Apan and Peterson 1998; Agarwal et al. 2005; Gorenflo et al. 2011). The problem is that the predicted probabilities of deforestation at the pixel scale determine the mean deforestation rate, that is, the intensity of deforestation at the landscape scale. As a consequence, when deforestation occurs, the progressive decrease in the mean distance to forest edge leads to a major increase in the mean deforestation rate at the landscape scale. Inversely, when deforestation occurs, the progressive increase in the mean elevation measurement can lead to a decrease in the deforestation rate at the landscape scale, even though the population density continues to increase. One possible way of overcoming this problem is to separate the process determining the intensity of deforestation (or “quantity” census Pontius and Millones 2011) from the process determining the location (or “allocation” census Pontius and Millones 2011) of the deforestation. This is the approach chosen by classic software that can be used to model and forecast deforestation, including CLUE-S (Verburg et al. 2002), Dinamica EGO (Soares et al. 2002), GEOMOD (Pontius et al. 2001), and Land Change Modeler (LCM) (Kim 2010). In the first step, these programs compute a “deforestation trend” by comparing land cover maps at two different dates. In the second step, they derive a transition potential map (per-pixel probabilities of shifting from a forest to a nonforest state, Eastman et al. (2005)) using different statistical methods and spatial factors. However, the “deforestation trend,” which determines the intensity of deforestation in the future, is usually a simple mean and is not related to dynamic explanatory variables such as population density (Mas et al. 2007). Consequently, it is impossible to forecast the effect of population expansion in developing countries on deforestation and the resulting carbon dioxide emissions using this statistic.

**Figure 1:**
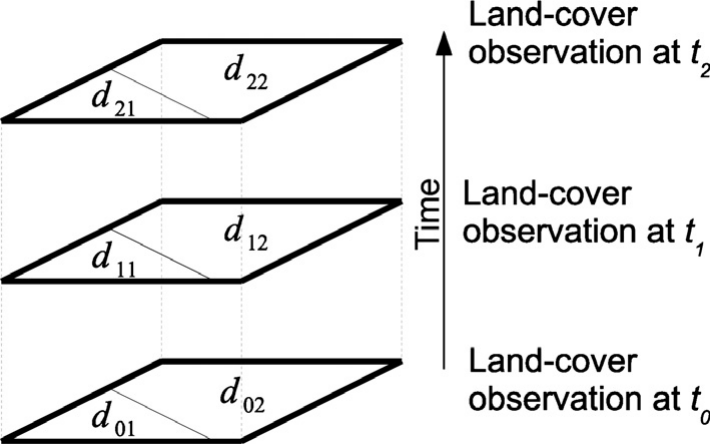
Satellite image mosaics and variable time interval between observations. We denoted *i* the rank of the land cover observation, *j* the index of the mosaic piece, and *d*_*ij*_ the date of the image for observation *i* and the mosaic piece *j*. For the analysis of large forest areas, adjacent satellite images (*ij* and *ij*^′^) are not necessarily available for the same date. Moreover, available satellite images acquired at particular dates may not be usable for analysis of land cover change because of a too dense cloud cover (≥10%). Thus, the time interval *T*_*i,i*+1*,j*_ between observations *i* and *i* + 1 for the mosaic piece *j* may differ from *T*_*i,i*+1*,j′*_ for the mosaic piece *j*^′^ and in the same way, *T*_*i,i*+1*,j*_ may differ from *T*_*i*+1*,i*+2*,j*_. This must be taken into account to avoid errors when estimating the mean deforestation rate (in%.year^−1^).

To accurately estimate the effect of population density on deforestation intensity, repeated observations of land cover change and population density are required over long periods of time and at large spatial scales (Ramankutty et al. 2007). For large forested areas, adjacent satellite images may not be available for the same date, and available satellite images acquired at the desired date may not be suitable for the analysis of land cover change if cloud cover is too dense (≥10%). For the same reasons, the time period for observations of land cover change might not be constant when using repeated observations over time. Consequently, the time interval for observations of land cover change can differ dramatically (by more than a year) from one observation to another (Fig. 1). To avoid serious errors, these differences in the time interval between land cover observations need to be taken into account when estimating the annual deforestation rate (Puyravaud 2003). This is not possible using the previously cited programs which estimate deforestation intensity by comparing land cover maps at two fixed dates (Pontius et al. 2001; Soares et al. 2002; Verburg et al. 2002; Kim 2010).

In this study, we present a coherent framework and new statistical tools to overcome these problems and to accurately forecast deforestation and the resulting carbon dioxide emissions while taking population expansion into account. As a case study, we used recent data on land cover changes covering two time periods from five sites in Madagascar's tropical humid and spiny-dry forests. Madagascar is widely known for its exceptional rates of both diversity and endemism in many taxonomic groups (Goodman and Benstead 2005), as well as for its low percentage of remaining native forest cover (Achard et al. 2002; Harper et al. 2007) and high level of threat associated with rapid population growth (Raftery et al. 2012). The method we present is simple, flexible, and overcomes the abovementioned problems. To encourage the use of this method, we provide a new R package (Ihaka and Gentleman 1996) named phcfM (for “programme holistique de conservation des forêts à Madagascar”), which includes functions for estimating the parameters of the demographic and deforestation models. We also provide associated R/GRASS scripts (Ihaka and Gentleman 1996; Neteler and Mitasova 2008), which outline the necessary steps for the modeling and forecasting procedures.

## Materials and Methods

### Definition of the study sites

**Figure 2:**
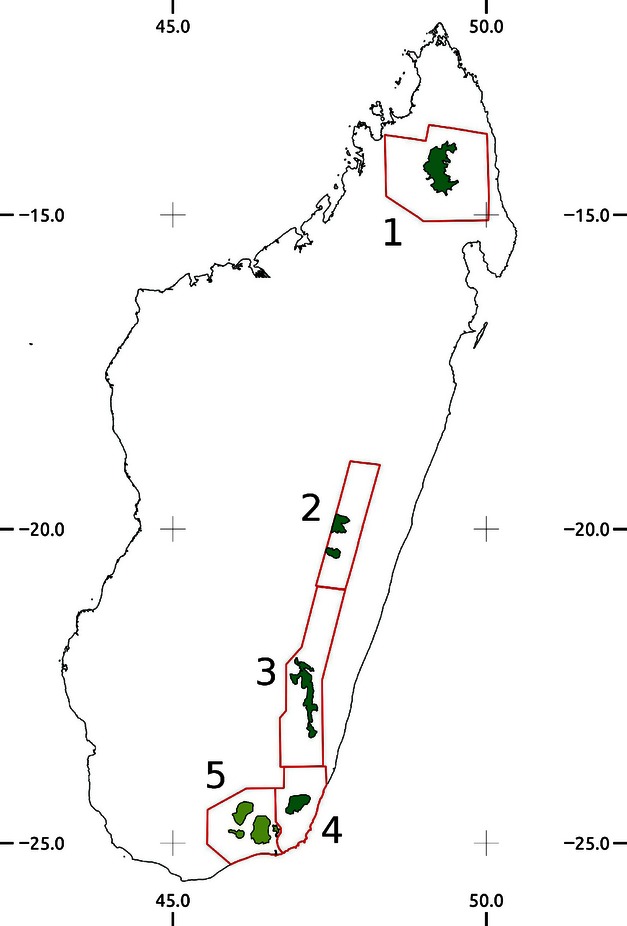
Location of study areas and project sites. The red line delimits the five study areas. The project sites within each study area are represented by colored polygons: dark green polygons for project sites in the humid forest and light green polygons for project sites in the spiny-dry forest.

The study focused on five areas in Madagascar (Table 1 and Fig. 2). Together, the study areas covered a total of 2,407,000 ha of tropical forest comprising 372,000 ha of spiny-dry forest (with precipitation <1000 mm.year^−1^) and 2,035,000 ha of humid forest (with precipitation ≥1000 mm.year^−1^). For each study area, the deforestation modeling approach (whose aim is to estimate parameters for the deforestation model) used data covering the whole study area. The deforestation and carbon dioxide emission forecasts (whose aim is to predict future deforestation and carbon dioxide emissions) were based on smaller project sites within each study area (Table 1 and Fig. 2). The study areas were selected based on the two following requirements. First, the study area had to be large enough to include sufficient data, in order to enable modeling of deforestation. Second, the deforestation process (intensity and location) had to be a priori homogeneous throughout the study area. The project sites corresponded to potential future protected areas defined by the Holistic Conservation Programme for Forests (HCPF) in Madagascar. The HCPF is a REDD+ pilot project implemented in the field by the GoodPlanet Foundation and the World Wildlife Fund (WWF).

**Table 1:** Study area and characteristics of the project sites

Id	Study area	Forest type	*S*_SA_	*F*_SA_	*S*_PS_	*F*_PS_	Date	ACD_PS_
1	Andapa	Humid	2610	1011	271	216	2008	88
2	Fandriana	Humid	1114	274	89	24	2010	40
3	Ivohibe	Humid	1839	490	179	113	2010	73
4	Fort-Dauphin I	Humid	887	260	83	54	2010	89
5	Fort-Dauphin II	Spiny-dry	1248	372	227	122	2010	16

The identifiers correspond to those in Figure 2. Size of study areas (*S*_SA_), project sites (*S*_PS_), forest cover in the study area (*F*_SA_), and forest cover in the project sites (*F*_PS_) are in thousands of hectares (×1000 ha). The sizes of the forest areas are for the year in the last column. The mean aboveground carbon density for the forest at the project site (ACD_PS_) is in Mg.ha^−1^ and was computed for the year 2010.

### Mapping the past deforestation using remote sensing techniques

For modeling the deforestation process, we needed observations of the past land cover change. We applied remote sensing techniques to obtain maps of past deforestation in the five study areas. For the remote sensing analysis, we used 30 × 30 m spatial resolution Landsat TM (Thematic Mapper) satellite images provided by the USGS (United States Geological Survey, Reston, VA) through GloVis (Global Visualization Viewer; Houska and Johnson 2012). Satellite images were selected with the aim of covering as much of the recent deforestation that occurred between 2000 and 2010. To obtain repeated observations of land cover change over time, we selected images for three different dates around 2000, 2005, and 2010. Depending on the availability of the Landsat TM images and due to the need to select images with the lowest possible cloud cover (*<*10%), we obtained a mosaic of satellite images for each study area at each date *t*_0_, *t*_1_, and *t*_2_ (Fig. S1) with different time intervals between observations (Figs. 1 and S1).

**Figure 3:**
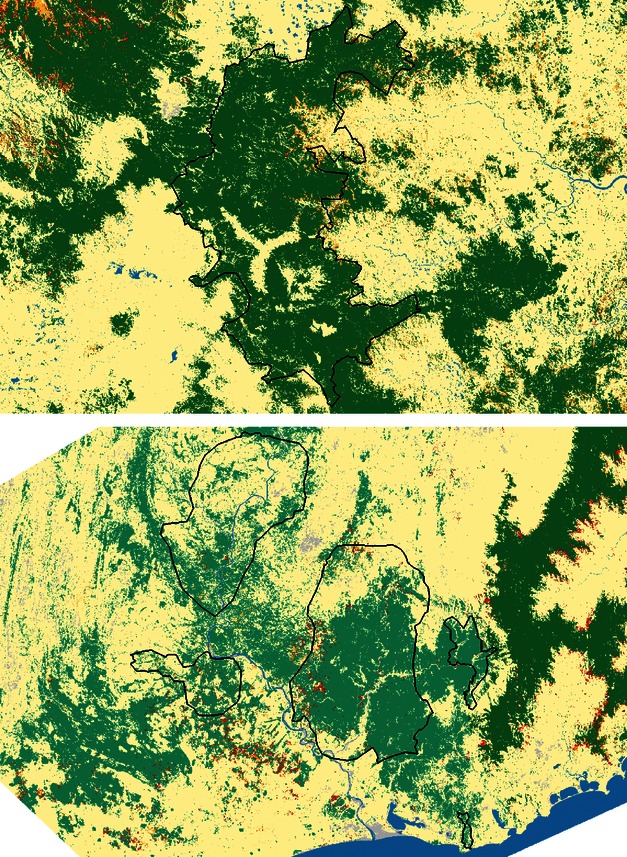
Historical deforestation at project sites 1 and 5 in the humid and spiny-dry forest. Patches of forest deforested between dates *t*_0_ and *t*_1_ are in orange, while patches of forest deforested between dates *t*_1_ and *t*_2_ are in red. Remaining forest is in dark green (humid forest) and light green (spiny-dry forest).

Using the satellite images at the three time points, a multi-date supervised classification of the land cover change was carried out following the methodology of Grinand et al. (C. Grinand, G. Vieilledent, F. Rakotomalala & R. Vaudry, in review). We used the Random Forests classification algorithm (Breiman 2001) available through R software (Ihaka and Gentleman 1996). Random forests efficiently manages the multi-modal spectral signatures associated with Landsat TM images acquired at several dates and in different seasons. We considered five classes of land cover: forest (class F), nonforest including rocks, crop land, and savanna (class P), wetland (class W), cloud (class C), and shade (class S). These were converted into seven classes of land cover change including deforestation (i.e., change in land cover from forest to nonforest) between dates *t*_0_ and *t*_1_ (class FPP), deforestation between dates *t*_1_ and *t*_2_ (class FFP) and unchanged land cover (FFF, PPP, CCC, SSS, and WWW). In the humid forest, the forest class was defined as 10% or more canopy cover for trees of 5 m or more in height. In the spiny-dry forest, the same definition was used except that the minimum height was set at 3 m. To build the classification trees, a training data set representative of the seven classes of land cover change was manually created by visual interpretation of the Landsat images. A number of additional data sets, including freely available QuickBird images from GoogleEarth™, and expert information were used as reference materials to help interpretation. In addition to the three TM spectral bands one (blue-green, 450–520 nm), four (near infrared, 770–900 nm), and five (mid-infrared, 1550-1750 nm), we used two spectral normalized indexes (the Normalized Difference Vegetation Index [NDVI] and the Normalized Infra Red Index [NIRI]) to build the regression trees from the training data set. The classification trees were then used to classify land cover changes and to obtain maps of past deforestation in all five study areas (Figs. 3 and S2).

### Demographic modeling from population census data

Using population census data in Madagascar, we built a demographic model that was used first, to estimate population density in the past and test the effect of population density on deforestation intensity and second, to project population density in the future and predict the future deforestation and carbon dioxide emissions. The more recent population census in Madagascar (RGPH: “recensement général de la population et de l'habitat”) was carried out in 1993 at the Firaisana (town) level. A more recent population census at the Fokontany (sub-town) level was carried out between 2004 and 2009. For both censuses, data were collected by the INSTAT (“Institut National de la Statistique à Madagascar”). Combining the two data sets for the Fokontanys covering our study areas (Fig. 2), we estimated a mean population growth rate using a statistical exponential model (eq. (1)). We denoted *P*_*k*_(*t*) the population of the Firaisana *k* at time *t* (in years, yr). We set *t* = 0 for the year 1993. We denoted *ρ* the annual population growth rate so that *dP*_*k*_(*t*)/*dt* = *ρP*_*k*_(*t*). We denoted *α*_0_ the mean population of a Firaisana for the year 1993. We added a random effect, *b*_*k*_, to account for the statistical dependence of the observations at the Firaisana level and to obtain a better estimate of the mean population growth rate *ρ* (Verbeke and Molenberghs 2009).


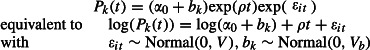
(1)

The Bayesian approach is particularly suitable for estimating the parameters of such a hierarchical mixed-effects model (Clark 2005). In this study, we chose this approach over the maximum likelihood approach. But from a technical point of view, all the modeling work could also be done using the maximum likelihood approach. We used noninformative conjugated priors with large variances: Inverse-Gamma (0.001, 0.001) for variances *V* and *V*_*b*_ and Normal(0, 1.0 × 10^6^) for parameters *α*_0_ and *ρ*. To do so, we used the function demography() available in the phcfM R package (see Appendices S5 and S6). We used the mean posterior of the population growth rate *ρ* to estimate the population *P*_*j*_(*t*) of any Fokontany *j* at time *t* using the data from the second population census at the Fokontany level.

### Modeling the intensity of deforestation from population density

For a better representation of the deforestation process, two sub-processes can be considered, a first one describing the intensity of deforestation and a second one describing the location of deforestation (Pontius and Millones 2011). In a first step, we modeled the process determining the intensity of deforestation. All the deforestation modeling was done for each study area separately. Following several previous studies on deforestation (Kaimowitz and Angelsen 1998; Agarwal et al. 2005; Gorenflo et al. 2011), we assumed that the intensity of deforestation depended on population density. We randomly sampled *Q*_0_ pixels covered by forest at date *t*_0_ and *Q*_1_ pixels covered by forest at date *t*_1_. These pixels were sampled outside the areas covered by clouds or shadows on the satellite images. Using a Normal approximation for the Binomial confidence interval at 95% and given a deforestation rate of about 1.0%.year^−1^ (Achard et al. 2002), a minimum number of 38,000 observations is necessary to estimate the intensity of deforestation with an uncertainty of less than ±0.1%.year^−1^. We set *Q*_0_ = *Q*_1_ = 20,000 and obtained a total of 40,000 observations. We denoted *Z* the random variable describing the deforestation process. We set *z*_*i*_ = 0 if the pixel *i* was still covered by forest and *z*_*i*_ = 1 if the pixel had been deforested during the time interval *Y*_*i*_ (in years). The random variable *Z* follows a Bernoulli distribution with probability *θ*_*i*_^′^ (eq. (2)). The parameter *θ*_*i*_^′^ was expressed as a function of the annual deforestation rate *θ*_*i*_ and of the time interval *Y*_*i*_ for pixel *i* (Puyravaud 2003). We tested the effect of the Fokontany population density *D*_*i*_ (in peop.km^2^) associated with pixel *i* on the intensity of deforestation *θ*_*i*_ using a logistic regression (eq. (2)). The population density *D*_*i*_(*t*) at date *t* for pixel *i* was computed using the demographic model assuming that every pixel in one Fokontany had the same population density equal to the population density at the Fokontany scale (*D*_*i*_(*t*)=*P*_*j*_(*t*)/area_j_ if *i* ∈ *j*).


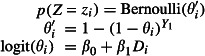
(2)

We estimated the model parameters in a hierarchical Bayesian framework using noninformative conjugated priors with large variances: Normal(0, 1.0 × 10^6^) for parameters *β*_0_ and *β*_1_. To do so, we used the function deforestation() available in the phcfM R package (see Appendices S5 and S6).

### Modeling the location of deforestation using spatial explicative factors

#### Potential spatial factors driving the location of deforestation

In a second step, we considered the sub-process determining the location of deforestation. The objective was to identify the factors explaining why deforestation was occurring at particular places. We modeled the probability of deforestation at the pixel level using several spatial factors that can be classified in different categories: (i) landscape factors, (ii) transport factors, (iii) socioeconomic factors, and (iv) land policy factors.

The landscape factors included the following: elevation (in m), forest fragmentation index, the shortest distance to forest edge (in m), and the shortest distance to previously deforested pixels (in m). The elevation data were obtained from the SRTM (Shuttle Radar Topography Mission). The fragmentation index was computed following the method of Riitters et al. (2000) who identified five forest classes: patch, transitional, perforated, edge, and interior. To compute the dynamic landscape factors that change with time (i.e., all landscape factors except elevation), we had to make the plausible assumption that the forest boundary did not significantly change between the dates of acquisition of the satellite images that made up the mosaic (Fig. S1).

The transport factors included the following: the shortest distance to the main road (in m) and the distance to the nearest main town in the Fivondronana (in m). The Fivondronana is an administrative entity grouping several Firaisana. Generally speaking, the main roads connect the main towns in each Fivondronana. These data were derived from the FTM maps (“Foiben-Taosarintanin'i Madagasikara”, Madagascar National Geographic Institute, http://www.ftm.mg).

The socioeconomic factors included the population density (in peop.km^2^) at the Fokontany level, which was obtained from the demographic model. Using data from the ILO project in Madagascar (Improve Public Information and Dialogue, http://www.ilo.cornell.edu), we also included the following potential explanatory variables defined at the Firaisana level: the percentage of poor, the number of mines, the number of cattle, and the percentage of farmers who use chemical fertilizers.

Land policy was described by a logical variable indicating whether the forest pixel *i* was located in a protected area. To determine this variable, we used the delimitation of the SAPM (“Système d'Aires Naturelles Protégées à Madagascar”) available at the Rebioma web-portal (http://www.rebioma.net/). We only used the protected areas managed by the ANGAP/MNP (Madagascar National Parks) which were created before 2003.

#### Statistical approach for modeling the location of deforestation

As for modeling the intensity of deforestation, we used a logistic regression to model the probability of deforestation at the pixel scale (eq. (3)). For the observations, we only selected the *Q*_1_ pixels between date *t*_1_ and date *t*_2_ in order to be able to compute the shortest distance to previously deforested pixels between date *t*_0_ and date *t*_1_. Explanatory variables were combined into a linear model. We denoted ***X***_***i***_ the vector of explanatory variables for pixel *i* and ***γ*** the vector of parameters. The probability of deforestation for each pixel *i* was given by the value of the latent variable *δ*_*i*_.


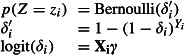
(3)

We estimated the model parameters in a hierarchical Bayesian framework using noninformative conjugated priors with large variances: Normal(0, 1.0 × 10^6^) for all the parameters of the vector ***γ***. Once more, we used the function deforestation(), which is available in the phcfM R package.

#### Importance of variables and model selection

To measure the importance of each factor in determining the spatial probability of deforestation, we compared the deviance of the full model (including all the spatial explanatory factors) with the deviance of the model that included all the spatial variables except the factor under consideration. The deviance of a model is defined as *D* = −2 log(*L*), *L* being the likelihood of the model (Spiegelhalter et al. 2002). The greater the difference in deviance between the two models, the more important the factor had in determining the probability of deforestation.

Using the full model, we also computed the credible interval of each parameter based on the 95% quantiles of the posterior distribution. If zero was included in this interval, the parameter was considered to not significantly differ from zero.

The approach we used to select variables for the final model was based on both statistical and empirical criteria. We selected the variables that were important in determining the probability of deforestation (positive gain in deviance) and whose effects were significantly different from zero. We rejected the variables whose effect was biologically inconsistent with the deforestation process (higher probability of deforestation in protected areas, e.g., Gorenflo et al. 2011).

### Forecasting deforestation using the demographic and deforestation models

#### Methodology used to forecast deforestation

Using the demographic model and the best deforestation model, we forecasted the deforestation in each study area independently. We ran simulations only for the potential REDD+ project sites in the study area, not for the whole study area. The time step used for the simulation was annual. The starting date (*s*_0_) for the simulation depended on the date of the latest satellite image for each study area. We forecasted the deforestation until year 2030. We combined the process determining the intensity of deforestation and the process indicating the spatial location of deforestation to forecast deforestation.

The first process determined the intensity of deforestation by computing a mean annual deforestation rate for the project sites from the model described by equation (2), which accounts for mean population density. The population of the Fokontanys at the project sites at date *s*_0_ was computed using the demographic model described by equation (1). The population density (in peop.km^2^) at the Fokontany level was computed taking into account the area (in km^2^) of the Fokontany. All pixels in a given Fokontany were considered to have the same population density. Then, the mean population density at the project site was estimated based on the forested pixels at the project site and was then used to compute the mean annual deforestation rate *θ*_0_ at date *s*_0_ at the project site. Given the total number of forested pixels at the project site, the annual deforestation rate *θ*_0_ was used to compute the number of pixels *n*_1_ likely to be deforested between date *s*_0_ and date *s*_1_.

The second process determined the spatial location of deforestation. Using the best model we estimated the probability of deforestation *δ*_*i*0_ for each forested pixel *i* at date *s*_0_ (eq. (3)). To do so, we needed to compute the landscape factors which varied with the forest delimitation (the shortest distance to forest edge and the fragmentation index) and with previous deforestation (the shortest distance to previously deforested pixels) at date *s*_0_. Every other spatial factor was assumed to remain constant with time at the pixel scale. Given the probability of deforestation *δ*_*i*0_ of each forested pixel, we simulated deforestation for the *n*_1_ pixels with the highest probability of deforestation.

The deforestation process, including the computation of the intensity of deforestation at the project site and the computation of the probability of deforestation for each forested pixel was repeated at each time step. Thus, we obtained maps of future deforestation between 2010 and 2030.

#### Model performance in forecasting deforestation

A cross-validation procedure was used to evaluate the performance of our approach in forecasting deforestation. For each study area, we divided the data set made up of the *Q*_1_ observations (from date *t*_1_ to date *t*_2_) into two, using 70% of the observations as training data set and 30% of the observations as test data set. We estimated the intensity of deforestation using the training data set in addition to the *Q*_0_ observations from date *t*_0_ to *t*_1_. We fitted the model for estimating the spatial probability of deforestation using the training data set with observations from date *t*_1_ to date *t*_2_ only, as we needed to compute the distance to previously deforested pixels. Models were used to predict deforestation based on the test data set. We computed a confusion table comparing the predictions of our modeling approach with the observations of the test data set. Several indices were computed from the confusion table (Appendix S7): overall accuracy (OA), the figure of merit (FOM), sensitivity, specificity, the true skill statistic (TSS), and Cohen's Kappa (Pontius et al. (2008), Liu et al. (2011)). The cross-validation procedure was repeated 10 times and the mean and standard deviation of each index was computed on the basis of the 10 repetitions.

### Forecasting carbon dioxide emissions

Forecasts of carbon dioxide emissions were obtained by overlaying maps of future deforestation with carbon maps associated with the aboveground forest biomass. Carbon maps (Figs. S3 and S4) of the Andapa and Fort-Dauphin (humid and spiny-dry forest) project sites were obtained from two previous studies (Asner et al. 2012; Vieilledent et al. 2012). Aboveground carbon density (ACD, in Mg.ha^−1^) was estimated for 83 forest plots based on tree diameter inventories (Asner et al. 2012) and biomass allometric models (Vieilledent et al. 2012). Airborne LiDAR (Light Detection and Ranging) data and remote sensing analysis of Landsat images were used to derive the carbon maps for the Andapa and Fort-Dauphin project sites. Altitude and the fraction of live photosynthetic vegetation (PV) classes were used as explanatory variables to estimate ACD at a resolution of 100 × 100 m (Asner et al. 2012). In this study, the same classes of altitude and NDVI were used to derive carbon maps for the Ivohibe and Fandriana project sites assuming that PV and NDVI were equivalent indexes (both indexes range between 0 and 1 and increase with an increase in the percentage of green live vegetation). For a forested pixel, data missing on the carbon map due to clouds or shadows on the Landsat image were replaced by an average value of the ACD for the forested area in the corresponding project site. To eliminate unrealistic impulsive noise (also known as “salt and pepper” noise) on the ACD maps, we smoothed the data spatially using a moving average window of 3 × 3 pixels. The carbon maps were obtained for the year 2010 (Asner et al. 2012). We used the year 2010 as the starting date for forecasting deforestation and CO_2_ emissions (Figs. 5, S5, and S6). We assumed no change in the carbon maps for the limited period (20 years) we used to forecast deforestation and CO_2_ emissions.

To forecast the CO_2_ emissions associated with deforestation at the project sites, we overlaid the future deforestation maps (Figs. 5, S5, and S6) with the carbon maps (Figs. S3 and S4). We used the ratio of the atomic mass of a CO_2_ molecule to the atomic mass of a carbon atom to compute the emission of CO_2_ (1 Mg of C = 44/12 Mg of CO_2_ equivalent).

## Results

### Intensity of the deforestation and population growth rate

**Figure 4:**
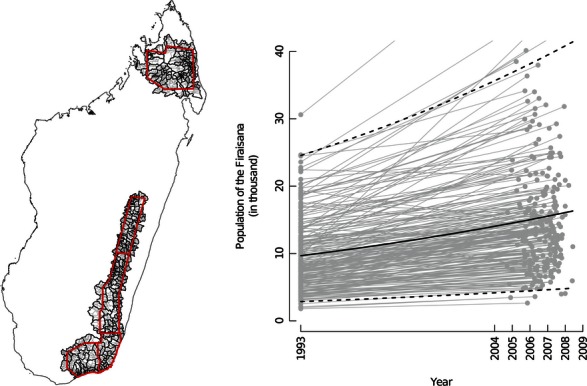
Demographic model from population census data. The exponential model was fitted on the population census data from the RGPH (“recensement général de la population et de l'habitat”) for Firaisana in 1993 combined with a more recent population census data acquired between 2004 and 2009 for Fokontany. In the figure on the left, the delimitation of Firaisana (black lines) and Fokontanys (gray lines) covering our study areas (red lines) are represented on the map of Madagascar. In the figure on the right, population growth in each Firaisana is represented by two gray dots connected by a gray line. The mean exponential growth population model is represented by a plain black line. The mean population growth rate was estimated to be 3.39%.year^−1^. The 95% confidence envelop including the Firaisana variability is represented by dashed black lines.

The mean annual deforestation rates varied considerably between the study areas, that is from 0.47%.year^−1^ for the Fort-Dauphin spiny-dry forest up to 2.45%.year^−1^ for the Fandriana humid forest. Using the Firaisana population census data for the five study areas and the exponential population growth model (eq. 1), we estimated an annual population growth rate of 3.39%.year^−1^ (Fig. 4). We found a significant positive effect of population density on the annual deforestation rate (Table 2), the latter being relatively homogeneous over the five study areas with values ranging from 0.010 to 0.026. The predicted increase in population density should lead to an increase in the annual deforestation rate in the long term. For example, considering the Ivohibe project site, the mean population density should increase from 14.18 peop.km^2^ in 2010 to 27.00 peop.km^2^ in 2030. Consequently, the intensity of deforestation should increase from 0.94%.year^−1^ in 2010 to 1.30%.year^−1^ in 2030. Because the population increase is exponential, the population density is increasing even more dramatically in areas that already have high population densities, leading to a marked increase in the intensity of deforestation (see the Fandriana study area in Table 2).

**Table 2:** Effect of population density on the annual deforestation rate

Id	Study area	*θ*_*p*1_	*θ*_*p*2_	*β*_0_	*β*_1_	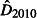	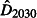	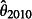	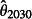
1	Andapa	0.87	0.98	−4.937	0.021^*^	8.65	16.48	0.85	1.00
2	Fandriana	2.40	2.45	−3.884	0.010^*^	37.28	70.99	2.94	4.11
3	Ivohibe	1.28	0.85	−5.032	0.026^*^	14.18	27.00	0.94	1.30
4	Fort-Dauphin I	1.16	1.20	−4.608	0.011^*^	18.16	34.57	1.20	1.42
5	Fort-Dauphin II	0.51	0.47	−5.773	0.022^*^	19.27	36.69	0.47	0.69

Analysis of past deforestation in the study area enabled a mean annual deforestation rate *θ* (in %.year^−1^) to be computed for period *p*_1_ (roughly 2000–2005) and *p*_2_ (roughly 2005–2010). Combining the model for the intensity of deforestation (which linked the annual deforestation rate *θ* and the population density *D* [in peop.km^2^]: logit(*θ*) = *β*_0_ + *β*_1_*D*), with the model of population growth with time (*dP*/*dt* = *ρP*, with *ρ* = 3.39%.year^−1^), we were able to estimate and forecast the mean population density (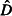) and the mean annual deforestation rate (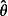) in 2010 and 2030 for the project sites. The effect of population density on the annual deforestation rate (parameter *β*_1_) in all the study areas was significantly different from zero (see the asterisk indicating that zero was outside the 95% confidence interval of the parameter) and of the same order of magnitude (∼0.02).

### Main spatial factors affecting the location of deforestation

Landscape variables appeared to be the main factors explaining the probability of deforestation (Table 3). Forest fragmentation was the main explanatory factor in study areas 3 and 4 in the humid forest, but was less explanatory in the spiny-dry forest. The distance to previously deforested patches was the main explanatory factor in study areas 1 and 2 in the humid forest and was also a strong explanatory factor in the spiny-dry forest. There was a marked decrease in the probability of deforestation with altitude in every study area in the humid forest, but this effect was not apparent in the spiny-dry forest where the landscape is much less mountainous. The distance to forest edge was relatively less important than other landscape factors, except in the spiny-dry forest. This may be due to the fact that the information associated with the distance to forest edge is already partly included in the fragmentation index. Regarding transport factors, the probability of deforestation was poorly explained by the distance to the main road whatever the study area, whereas the distance to the main town was a strong explanatory factor in the spiny-dry forest and in study areas 2 and 3 in the humid forest (Table 3). There was a lower probability of deforestation in protected areas in study areas 3 and 4, but this effect was weaker than other spatial factors and was not observed in study areas 1 and 5 (Table 3). This is probably due to the fact that landscape factors mask the effect of the land policy as protected areas are often located at higher altitudes, and in places where the forest is less fragmented. Regarding socioeconomic factors, we only used the effect of the population density for study areas 3 and 4 in the final models. With a few exceptions, the other socioeconomic factors (the percentage of poor, the number of mines, the number of cattle, and the percentage of farmers using chemical fertilizers at the Firaisana level) generally had a very weak effect on the probability of deforestation. Other disadvantages of these socioeconomic factors are that they are difficult to predict and that they were obtained at a coarse spatial resolution at the Firaisana level. We consequently decided not to use them in the final model we used to forecast deforestation.

**Table 3:** Importance and selection of variables

	SA1			SA2			SA3			SA4			SA5		
				
Variable/SA	I	S	V	I	S	V	I	S	V	I	S	V	I	S	V
Intercept		N^*^	−1.9868		P	0.0569		N^*^	−0.7740		N^*^	−1.4409		N^*^	−2.1219
Landscape															
frag. index	46			524			212			23			9		
trans.		N	−0.2122		N^*^	−0.6513		N^*^	−0.7965		N	−0.2172		N	−0.2941
perf.		N^*^	−0.4928		N^*^	−1.0662		N^*^	−1.4010		N^*^	−0.4284		N^*^	−0.5822
edge		N^*^	−0.6215		N^*^	−1.3934		N^*^	−1.7412		N	−0.4058		N	−0.4431
int.		N^*^	−1.1724		N^*^	−1.7805		N^*^	−2.0465		N^*^	−0.5060		N^*^	−0.6839
dist. dpatch	140	N^*^	−3.78E-3	165	N^*^	−2.06E-3	22	N^*^	−9.58E-4	584	N^*^	−2.26E-3	105	N^*^	−3.01E-4
alt.	78	N^*^	−9.88E-4	75	N^*^	−8.79E-4	40	N^*^	−1.05E-3	75	N^*^	−1.18E-3	24	P^*^	
dist. fedge	0	P		7	P^*^		82	N^*^	−6.89E-3	45	N^*^	−5.45E-3	34	N^*^	−3.11E-3
Transport															
dist. road	0	N		2	P		3	P^*^		6	N^*^	−6.20E-6	29	P^*^	
dist. town	0	N		188	N^*^	−2.57E-5	26	N^*^	−2.26E-5	0	P		305	N^*^	−4.85E-5
Land policy															
prot. area	2						8			7			0		
pres.		P^*^						N^*^	−0.3081		N^*^	−0.4199		P	
Socioeconomy															
pop. dens.	13	N^*^		53	N^*^		9	P^*^	3.66E-3	4	P^*^	3.58E-3	9	N^*^	
perc. poor	12	N^*^		5	N^*^		15	N^*^		4	N^*^		5	P^*^	
nb. cattle	1	N		36	P^*^		4	N^*^		6	P		27	N^*^	
nb. mines	4	N^*^		9	N^*^		12	N^*^		0	P		6	N^*^	
perc. chem	1	N		29	N^*^		1	N		16	P^*^		23	N^*^	

For each study area (from SA1 to SA5), we estimated the relative importance (column I) of the variables in determining the probability of deforestation. The importance of a variable is expressed in points of deviance gained when the variable is included in the model. Column S indicates the sign (N for negative, P for positive) of the effect of each variable and an asterisk indicates that the effect is significantly different from zero at a threshold of 5%. Column V lists the parameter values for the variables selected in the final model.

### Model performance in forecasting deforestation

The models performed well in forecasting deforestation in all five study areas. Table 4 lists the mean values of the five performance indices. The mean overall accuracy (OA) was very high (≥0.85) indicating a high probability of correctly predicting either deforestation or absence-of-deforestation. Similarly, specificity (Spe), which indicates the probability of correctly predicting the absence-of-deforestation, was also very high (≥0.92). However, due to the low deforestation rates (∼1%.year^−1^), a less reliable model that predicted no deforestation would also have high overall accuracy and high specificity. It was consequently necessary to use other indices that reflect the probability of correctly predicting deforestation pixels. The figure of merit (FOM), sensitivity (Sen), the true skill statistic (TSS), and Cohen's Kappa (K) were greater than 0.14, 0.31, 0.24, and 0.21, respectively, for models in the humid forest. The values of the indices in the spiny-dry forest were lower than those in the humid forest (Table 4), indicating that the model was less efficient in describing the process of deforestation in the spiny-dry forest than in the humid forest. Nevertheless, the indices were sufficiently high in the spiny-dry forest to enable realistic forecasts of deforestation.

**Table 4:** Performance of the model in forecasting deforestation

Id	Study area	OA	FOM	Sen	Spe	TSS	K
1	Andapa	93 (0.2)	14 (1.4)	41 (3.4)	94 (0.1)	35 (3.5)	21 (2.2)
2	Fandriana	85 (0.3)	20 (1.0)	31 (1.2)	93 (0.2)	24 (1.4)	25 (1.5)
3	Ivohibe	94 (0.3)	23 (2.4)	41 (2.9)	97 (0.2)	38 (3.0)	34 (3.2)
4	Fort-Dauphin I	93 (0.3)	22 (1.6)	32 (2.1)	97 (0.1)	29 (2.2)	32 (2.2)
5	Fort-Dauphin II	96 (0.1)	10 (1.1)	18 (1.9)	98 (0.1)	16 (2.0)	16 (1.9)

Six performance indices were computed: overall accuracy (OA), figure of merit (FOM), sensitivity (Sen), specificity (Spe), true skill statistic (TSS), and Cohen's Kappa (K). A cross-validation procedure in which the data set was divided into training data (70%) and test data (30%) was used to compute the indices. The table lists the mean values and standard deviation of the indices for 10 repeated cross-validations.

### Amount of CO_2_ emissions associated with deforestation

**Figure 5:**
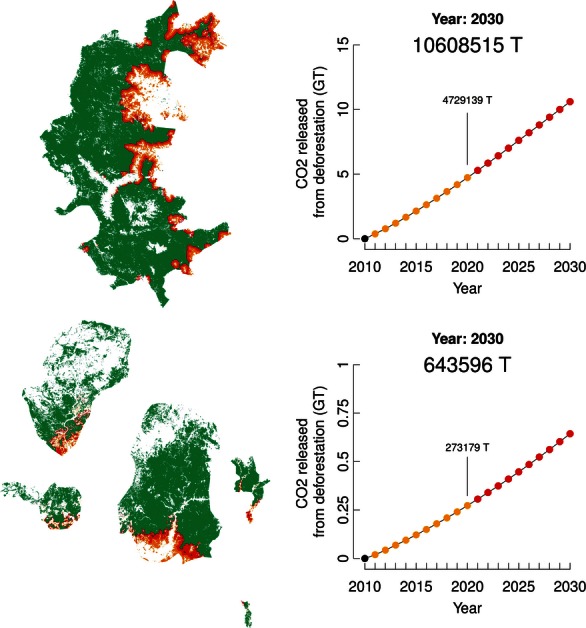
Forecast of anthropogenic deforestation and carbon dioxide emissions. Forecasts are shown for the Andapa project site in the humid forest (top figure) and for the Fort-Dauphin II project site in the spiny-dry forest (bottom figure). Patches of deforestation and carbon dioxide emissions for the period 2010–2020 are in orange and for the period 2020–2030, in red. The forest remaining in 2030 is in dark green. Carbon dioxide emissions correspond to the loss of aboveground biomass due to deforestation and do not include belowground biomass or soil carbon.

Figures 5, S5, and S6 show the deforestation forecasts for 2010–2030 for the five study areas with their associated CO_2_ emissions. Comparing the deforestation forecast with past deforestation (Figs. 3 and S2), future deforestation would be expected near places where deforestation was already high in the past. The amount of CO_2_ emissions associated with deforestation was mainly explained by two processes. First, it depended on the level of ACD in future deforestation areas. For example, in the Fort-Dauphin humid forest, CO_2_ emissions should increase exponentially (Fig. S6) because deforestation will spread toward mid-elevation areas with much higher ACD (Fig. S3). This result highlights the importance of spatializing both ACD and deforestation to obtain accurate predictions of CO_2_ emissions associated with deforestation. Second, the amount of CO_2_ emissions depended on the annual deforestation rate. The low level of CO_2_ emissions after 20 years of deforestation in the spiny-dry forest (Fig. 5) was due both to a low mean ACD (16 Mg.ha^−1^, Table 1) and to a low mean annual deforestation rate (0.49%.year^−1^, Table 2). The increase in the annual deforestation rate associated with the population growth rate led to an increase in the amount of CO_2_ emitted each year and contributed to the exponential emissions of CO_2_ with time (Fig. S6 and S7). For the 407,000 hectares of humid forest in the project sites in 2010, a total of 18,201,512 tons of CO_2_ should be emitted between year 2010 and year 2030, corresponding to 2.24 T.ha^−1^.year^−1^. For the spiny-dry forest project site, the emission rate should be 0.26 T.ha^−1^.year^−1^.

## Discussion

### Comparison with other approaches used to model and forecast deforestation

Statistical methods and predictive performances of the programs which can be used to forecast deforestation (CLUE-S, Dinamica EGO, GEOMOD and LCM) were compared in several recent studies (Mas et al. 2007; Kim 2010; Pérez-Vega et al. 2012). Here, we briefly present the statistical and practical advantages of our approach over these programs. In this study, we used logistic regressions to model both the intensity of deforestation and the location of deforestation. Logistic regression has the advantage of being simple and is frequently used in ecology to handle binary data (Hilborn and Mangel 1997). Thus, our approach should not involve any serious problems of understanding regarding the statistical assumptions and the interpretation of the model parameters. In comparison, the Multilayer Perceptron (MLP) model proposed by LCM is often described as a black box, which makes incorporation of expert knowledge rather difficult. It is also not suitable for multi-scenario prospective modeling as the relationship between the explanatory variables and deforestation cannot be easily modified (Kim 2010; Pérez-Vega et al. 2012). Moreover, the performance of the MLP in predicting deforestation was no better than the logistic regression in the study by Kim (2010). Another advantage of logistic regression is that the model is flexible: explanatory variables can be continuous or categorical and nonlinear relationships between variables and the deforestation rate can be estimated using polynomials. In comparison, Dinamica EGO and GEOMOD need to transform the continuous covariates into categorical data to compute the transition probability maps. Although logistic regression is available in CLUE-S and in LCM, it is only to model the location of deforestation, not the intensity of deforestation. Because population density is a key factor for the intensity of deforestation in developing countries (Allen and Barnes 1985; Kaimowitz and Angelsen 1998; Pahari and Murai 1999; Geist and Lambin 2001; López-Carr 2004; Agarwal et al. 2005; López-Carr et al. 2005), it is important to include this variable in the model and to modify the intensity of deforestation as a function of population growth. None of the available programs offers this possibility (Mas et al. 2007). Our approach also includes a step for model selection where both statistical criteria and empirical knowledge can be used to select the variables to be included into the model. In this sense, our approach is similar to the one proposed in Dinamica EGO where the weights of evidence can be edited. The incorporation of expert knowledge can corroborate or contradict the purely statistical approach in order to obtain a more realistic model. In our method, the use of scripts for the R (Ihaka and Gentleman 1996) and GRASS GIS (Neteler and Mitasova 2008) open-source software ensures maximum flexibility (see Appendix S5 and S6). The use of these programs is facilitated by the existence of numerous educational tutorials (Crawley 2007; Neteler and Mitasova 2008). Moreover, minimal changes should be required to adapt the scripts to the different contexts found in the tropical world. Another advantage of our approach is that deforestation analysis can be performed for large forest areas, that is at the sub-national or national scale (whereas CLUE-S software is more appropriate for small regional areas for example) and for a long period of time with repeated land cover observations. This is possible for two reasons. First, our approach takes advantages of the computational efficiency of R and GRASS GIS. Second, the phcfM R package we developed allows the parameters of a logistic regression model to be estimated taking into account the variable time intervals between land cover observations (see Appendices S5, S6, and S8). Handling such data is not possible when using the four programs cited above.

### Baseline deforestation scenarios for the REDD+ Programme

One of the most challenging aspects in designing the REDD+ Programme is estimating the baseline scenarios (Obersteiner et al. 2009). These scenarios describe the amount of CO_2_ emissions for a particular forest area under “business-as-usual” development. By describing the future emission pathway without any conservation and development measures, baseline scenarios are crucial for determining the success in reducing deforestation and CO_2_ emissions (Olander et al. 2008). Using our approach, we obtained relatively high model performance in comparison with other studies and we reduced the uncertainty associated with the baseline scenario. The FOM was greater than or equal to 14% in the humid forest and 10% in the spiny-dry forest. In a study analyzing 13 models of land change, Pontius et al. (2008) showed that the FOM was strongly positively correlated with the observed net change (indeed, the lower the deforestation rate, the more difficult it is to forecast the exact location of the deforested pixels). When the net change was ≤2.5%, the FOM was ≤7.5% and when the net change was ≤10%, the FOM was ≤15%. Kim (2010) also compared the values of the FOM obtained using GEOMOD and LCM software in a deforestation study in Bolivia: the highest value of the FOM he obtained was 8%. In this study, for equivalent net change values, we obtained much higher FOM values. The relatively good performance of our model was confirmed by the values obtained for the TSS (from 0.16 to 0.38) and the Kappa statistics (from 0.16 to 0.34). In comparison, using GEOMOD software in Mexico, Guerrero et al. (2008) found lower Kappa values (from −0.03 to 0.29). Finally, we estimated mean CO_2_ emissions of 2.24 and 0.26 T.ha^−1^.year^−1^, respectively, for the humid and spiny-dry forests of Madagascar. Reducing the uncertainty associated with baseline scenario is essential for financial efficiency given the limited resources dedicated to the REDD+ Programme and also to avert the risk of artificial inflation of avoided deforestation and CO_2_ emissions (Obersteiner et al. 2009). Olander et al. (2008) presented the essential characteristics of a good method for establishing baseline scenarios including feasibility, accuracy, transparency, comprehensiveness, and flexibility. Here, we present such a method using open-source and free software, while sharing data and computer scripts so that our study can be easily extended to the rest of Madagascar and to other tropical developing countries.

### Association between people and deforestation in Madagascar

Estimated deforestation rates ranged from 0.47%.year^−1^ for the Fort-Dauphin spiny-dry forest to 2.45%.year^−1^ for the Fandriana humid forest. These regional deforestation rates are of the same order of magnitude compared to previous estimates for the whole of Madagascar. Achard et al. (2002) estimated annual deforestation rates of between 1.4% and 4.7% for Madagascar for the period 1990–1997 and Harper et al. (2007) estimated rates of 0.8%, 0.7%, and 1.2% for humid, spiny, and dry forest, respectively, for the period 1990–2000. These values identify Madagascar as one of the hot spots of deforestation compared to other tropical countries (Achard et al. 2002). This study shows that the deforestation rates vary considerably from one region to another and that the use of sub-national deforestation models is preferable to a national model.

While estimating the parameters of the deforestation intensity model, we found a significant positive effect of population density on the annual deforestation rate. The population density effect was relatively homogeneous over the five study areas with values ranging from 0.010 to 0.026. The increasing intensity of deforestation that accompanies increasing population density is usually hard to estimate if the population density is associated with other explanatory variables in statistical models. For example, Gorenflo et al. (2011) and Apan and Peterson (1998) found that the effects of population density were generally of limited statistical significance and of low magnitude in Madagascar and in the Philippines. This could be explained by the fact that the population density is often observed at a coarser resolution than other explanatory variables such as landscape variables (elevation, distance to forest edge, distance to roads and towns, etc.). One possible way to overcome this problem is to use hierarchical Bayesian spatial models to account for heterogeneity in data resolution (Agarwal et al. 2005). Nevertheless, fitting such models is computationally much more demanding than fitting more standard models and such models are not easily accessible to the scientific community. As shown in this study, another possible approach is to separate the process determining the intensity of deforestation from the process determining the location of deforestation and to include the population density in the first process. Using this approach, we were able to estimate a significant effect of population density on the intensity of deforestation with a rather high magnitude for our study areas. Using hierarchical Bayesian spatial models, Agarwal et al. (2005) also found a significant effect of population density on the probability of deforestation. This result was previously observed at the global scale using simple correlation models (Pahari and Murai 1999). Even though the relationship between population density and deforestation may be complex depending on the socioeconomic and political context, as outlined by Gastineau and Sandron (2006) and Geist and Lambin (2001), the significant effect of population density on deforestation in poor developing tropical countries, where people's livelihoods depend to a great extent on forest resources, can hardly be questioned.

For the five study areas in Madagascar, we estimated an annual population growth rate of 3.39%.year^−1^. This regional result is slightly above the national population growth estimated by the United Nations which places Madagascar in the top 25 of the countries with the highest population growth rate, with a value of 2.66%.year^−1^ for the period 2010–2015 (United Nations 2011). This suggests that the population of Madagascar will double in the next 25 years, that is, from 21 million in 2011 to about 40 million in 2045. Concerning our five study areas, the high population growth rate (3.39%.year^−1^) should lead to a significant increase in the annual deforestation rate of more than 1%.year^−1^ between 2010 and 2030 in densely populated areas. To conclude, based on the example of Madagascar, we would like to emphasize the risk of an increase in the speed of deforestation in the short term in tropical developing countries facing rapid demographic expansion. The risk is particularly high for Africa (United Nations 2011; Raftery et al. 2012) where the total population will probably not start to decrease before 2050 (Raftery et al. 2012).

## Supporting Information

**Appendix S1.** Acquisition dates of the satellite images.

**Appendix S2.** Additional deforestation maps.

**Appendix S3.** Aboveground carbon density maps.

**Appendix S4.** Additional deforestation forecasts.

**Appendix S5.** The phcfM R package for modeling population growth and deforestation.

**Appendix S6.** R/GRASS scripts for modeling and forecasting deforestation.

**Appendix S7.** Mathematical formulas for model performance indexes.

**Appendix S8.** Comparison with Land Change Modeler and Dinamica EGO.
