# Supplementary material for: Forecasting deforestation and carbon emissions in tropical developing countries facing demographic expansion: a case study in Madagascar
Source: Ecol Evol. 2013 May 3;3(6):1702–16. doi: 10.1002/ece3.550 (PMC3686203; doi:10.1002/ece3.550)
Supplement: Supplementary file 1 [file ece30003-1702-SD1.pdf]

Forecasting deforestation and carbon emissions in tropical developing countries facing demographic expansion: a case study in Madagascar

SUPPORTING INFORMATION

Ghislain Vieilledent[⋆,1,2] Clovis Grinand[3]
Romuald Vaudry[3]

[⋆] **Corresponding author:** \E-mail: ghislain.vieilledent@cirad.fr \Phone: +261.(0)32.07.235.34 \Fax: +261.(0)20.22.40.821
[1] **Cirad** – UPR BSEF, F34398 Montpellier Cedex 5, France
[2] **Cirad-Madagascar** – DRP Forêt et Biodiversité, BP 853, Ambatobe, 101-Antananarivo, Madagascar
[3] **GoodPlanet** – Fondation GoodPlanet, Domaine de Longchamp, 1 carrefour de Longchamp, F-75116 Paris, France

1 Appendix 1: Acquisition dates of the satellite images

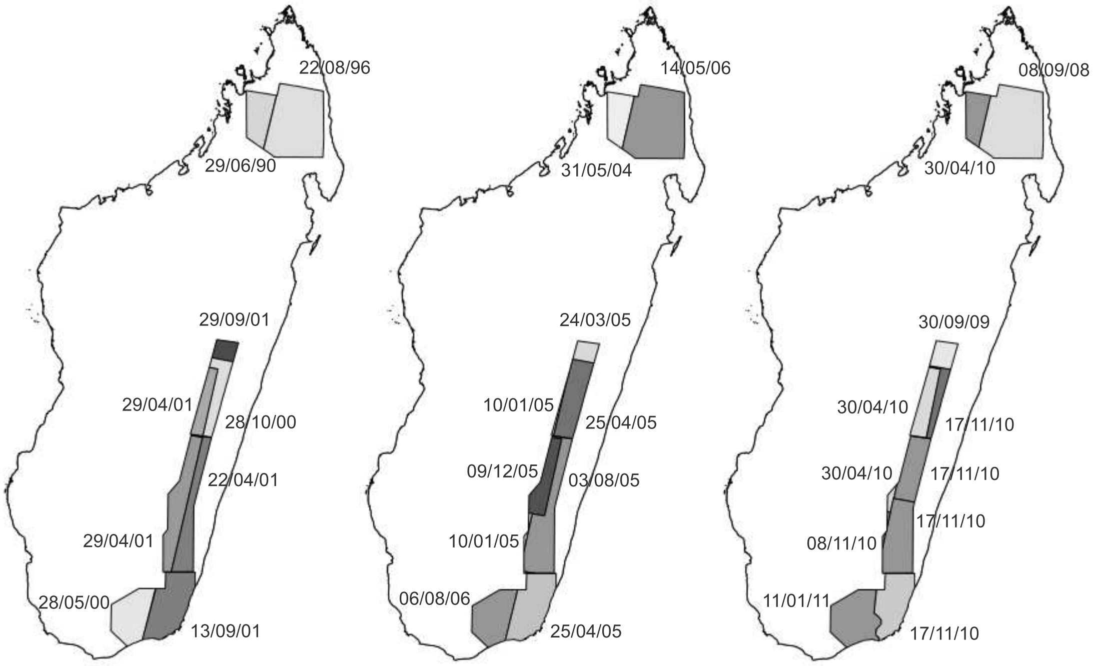

Figure S1: **Acquisition dates of the satellite images used for the analysis of land cover change.**

2 Appendix 2: Additional deforestation maps

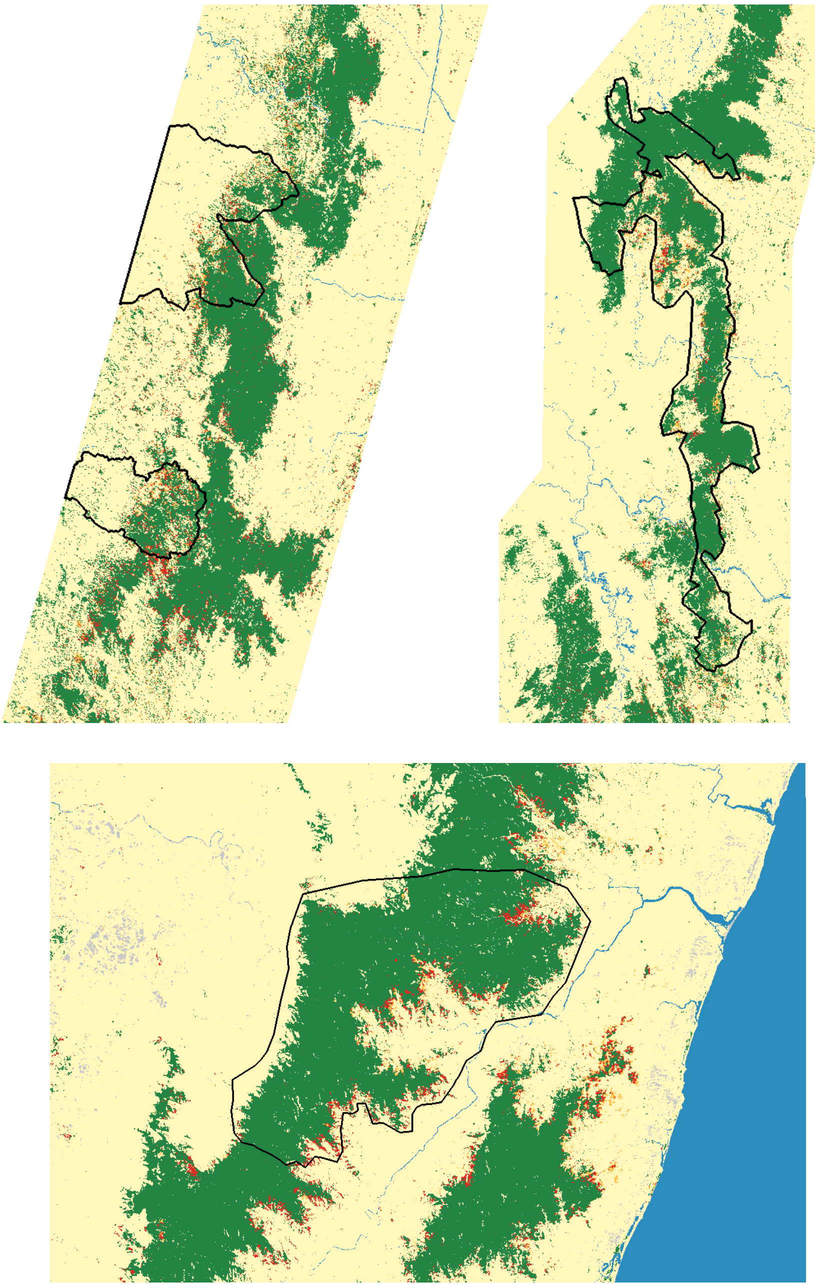

Figure S2: **Historical deforestation at project sites 2, 3 and 4 in the humid forest.** Patches of forest deforested between date t_0 and t_1 are in orange; patches of forest deforested between date t_1 and t_2 are in red. Remaining humid forest is in dark green.

3 Appendix 3: Aboveground carbon density maps

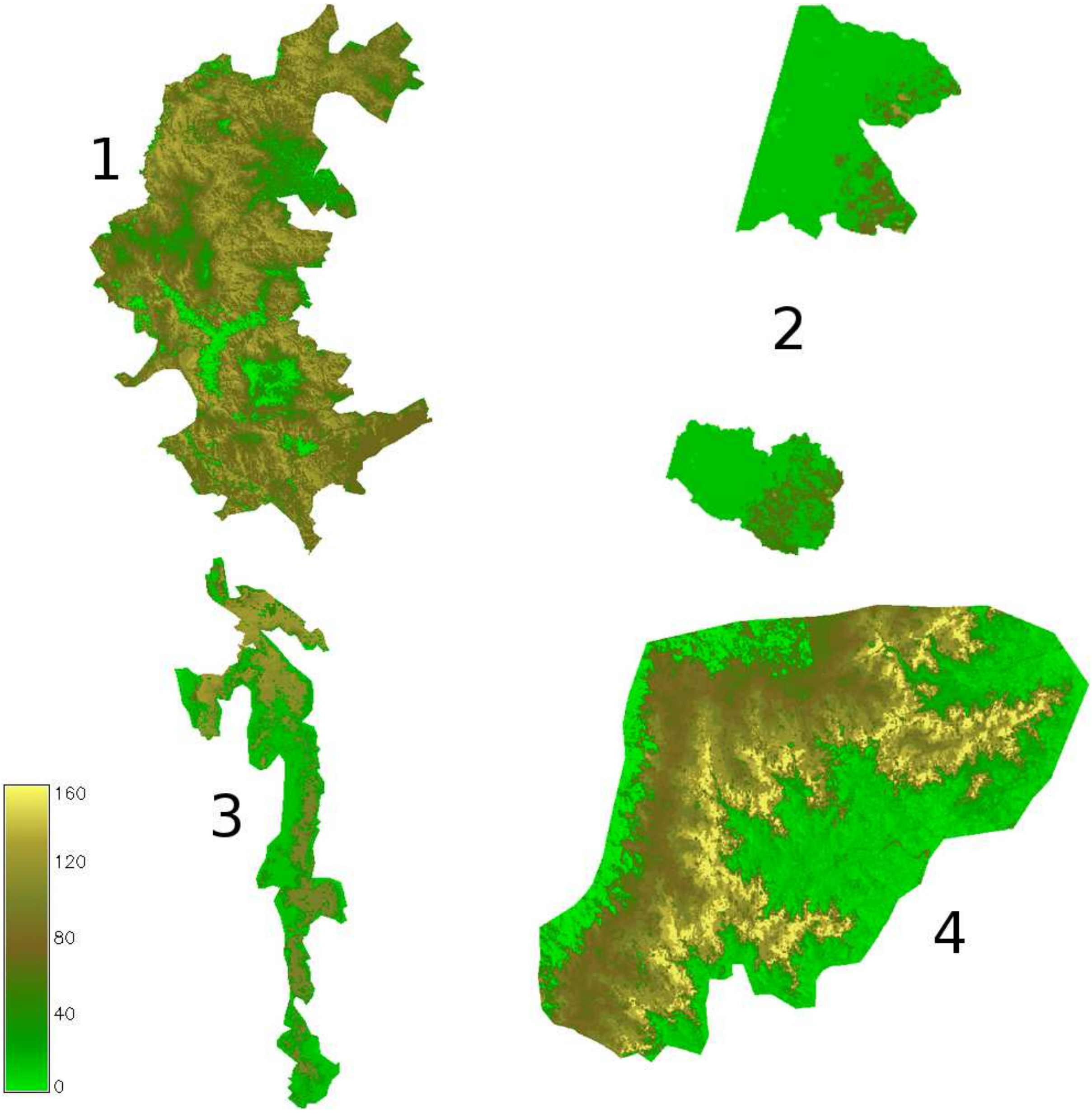

Figure S3: **Carbon maps of the project sites in the humid forest.** Aboveground carbon density (ACD) is in Mg.ha^{-1}. For each project site, the scale was optimized for better visualization of the ACD gradient.

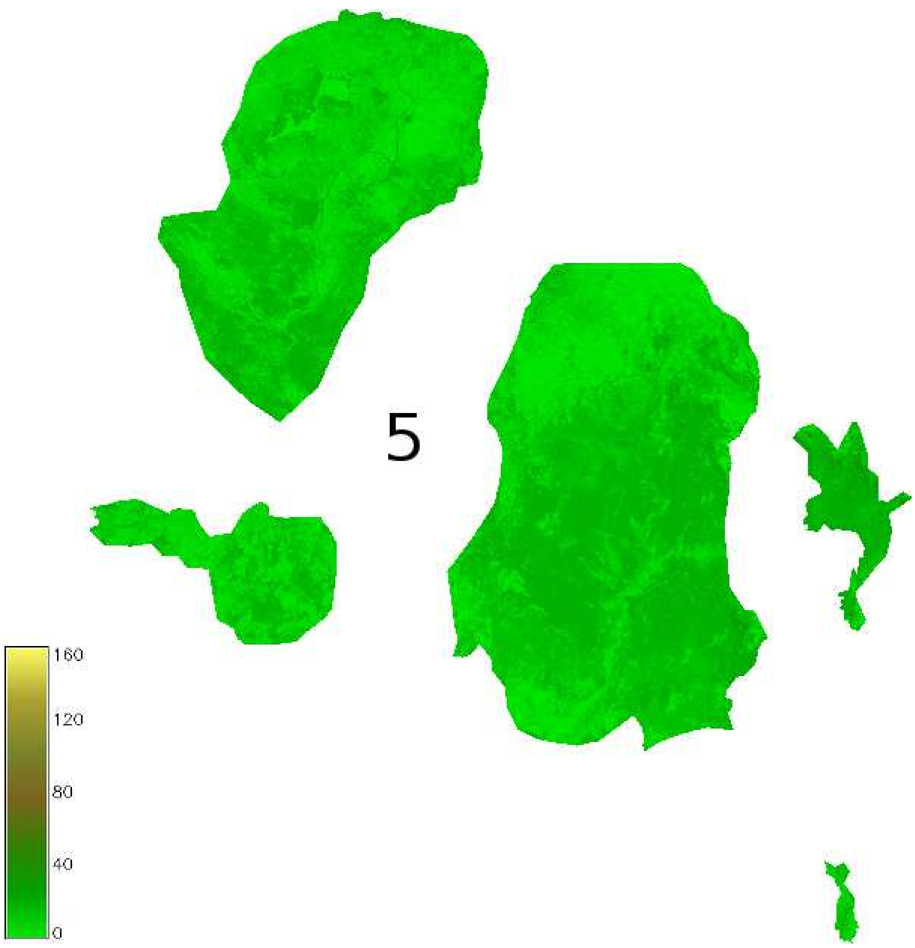

Figure S4: **Carbon maps of the project site in the spiny-dry forest.** Aboveground carbon density (ACD) is in $Mg.ha^{-1}$.

4 Appendix 4: Additional deforestation forecasts

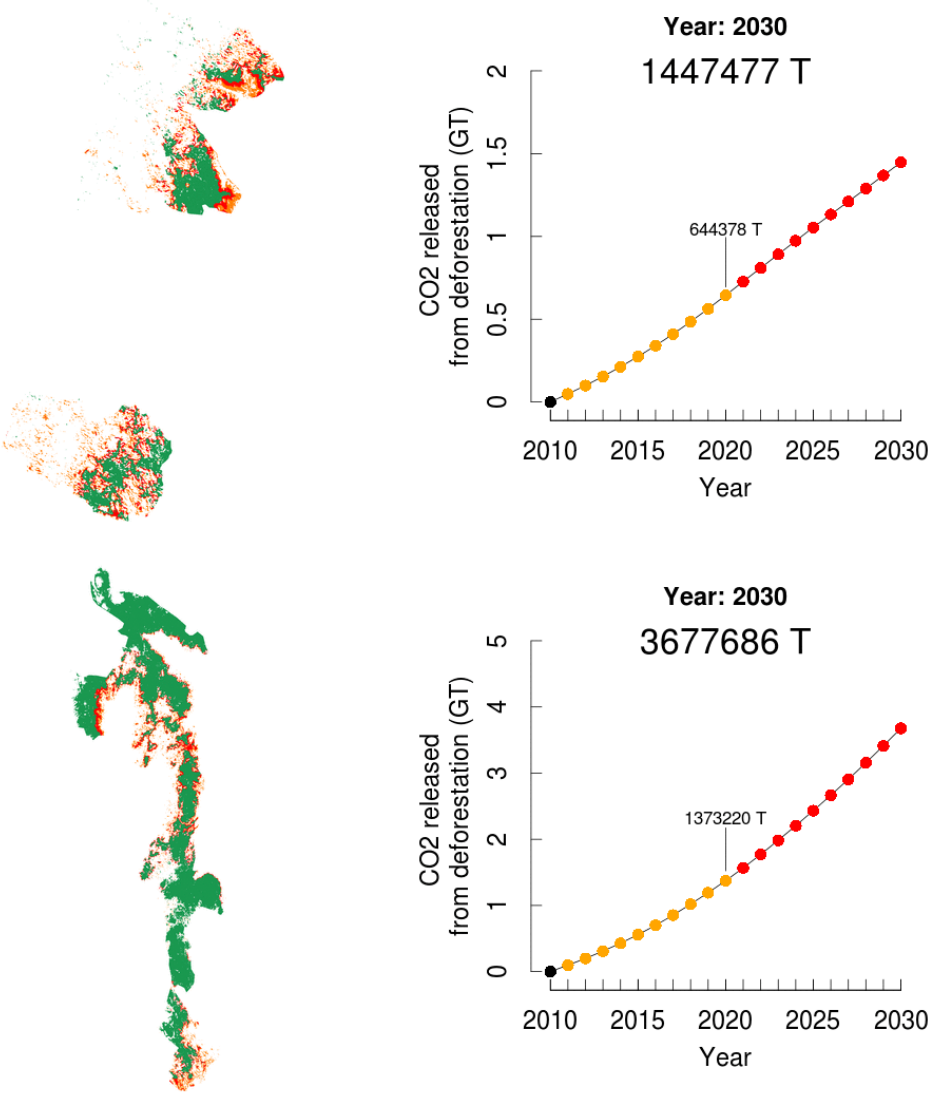

Figure S5: **Forecast of anthropogenic deforestation and carbon dioxide emissions.** Forecasts are given for the Fandriana (top figure) and Ivohibe (bottom figure) project sites in the humid forest. Patches of deforestation and carbon dioxide emissions are in orange for the period 2010–2020 and in red for the period 2020–2030. Forest remaining in 2030 is in dark green. Carbon dioxide emissions correspond to the loss of aboveground biomass due to deforestation and do not take belowground biomass or soil carbon into account.

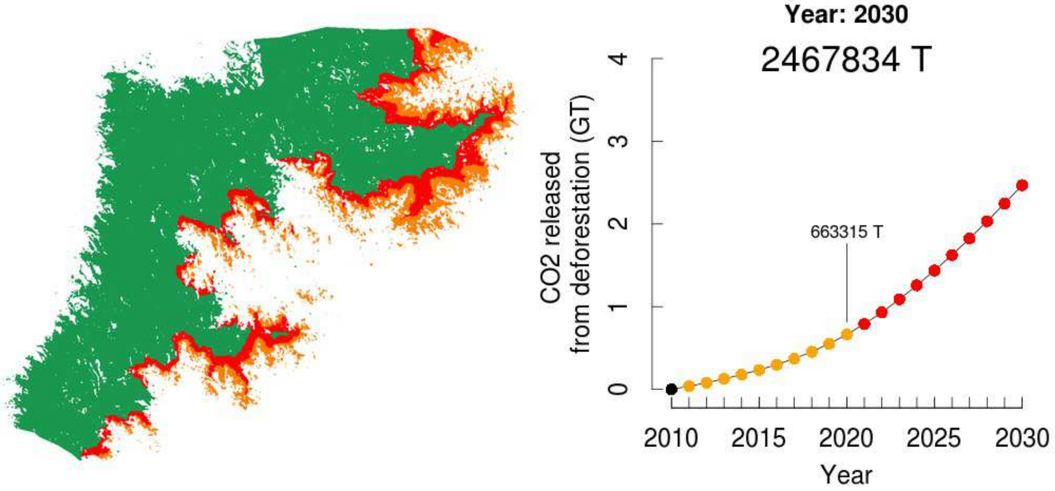

Figure S6: **Forecast of anthropogenic deforestation and carbon dioxide emissions.** Forecasts are shown for the Fort-Dauphin I project site in the humid forest. Patches of deforestation and carbon dioxide emissions are in orange for the period 2010–2020 and in red for the period 2020–2030. The remaining forest in 2030 is in dark green. Carbon dioxide emissions correspond to the loss of aboveground biomass due to deforestation and do not take belowground biomass or soil carbon into account.

5 Appendix 5: The phcfM R package for modelling population growth and deforestation

phcfM is an R package for modelling anthropogenic deforestation. It is named after the REDD+ pilot-project *"programme holistique de conservation des forts à Madagascar"*. It includes two main functions: (i) demography(), to model population growth with time in a hierarchical Bayesian framework using population census data and Gaussian linear mixed models and (ii) deforestation(), to model the deforestation process in a hierarchical Bayesian framework using land cover change data and Binomial logistic regression models with variable time intervals between land cover observations.

The two functions use embedded Gibbs samplers written in C++ with the Scythe statistical library (Pemstein *et al.*, 2011) to reduce computation time. The demography() function uses Algorithm 2 from Chib & Carlin (1999) to efficiently sample the posterior distributions of a hierarchical Gaussian linear regression model. The demography() function is equivalent to the MCMChregress() function recently added to the MCMCpack R package written by Martin *et al.* (2011). The deforestation() function uses an adaptive Metropolis algorithm to efficiently sample the posterior distributions of a Binomial logistic regression model with variable time intervals between censuses. This function is an improvement on the MCMClogit() function available in MCMCpack, as the latter does not include adaptive algorithms or manage variable time intervals between censuses. The two functions, demography() and deforestation(), were combined in a single R package to facilitate the modelling and forecast of anthropogenic deforestation. Source code for Linux, binaries for Windows and Mac OS X, and package manual are available on the phcfM R package website: http://phcfM.sf.net.

6 Appendix 6: R/GRASS scripts for modelling and forecasting deforestation

A GRASS location (phcfM_SM) and two mapsets (PERMANENT and study_area_4) with geographical data layers are available to illustrate the method used in this study for the study area and project site #4. Associated with the GRASS location, a folder (./scripts) includes the data, the R/GRASS scripts used for the demographic and deforestation models and output folders. The following scheme illustrates the structure of the ./scripts folder. The phcfM_SM GRASS location and the ./scripts folder are also available on the phcfM R package website: http://phcfM.sf.net.

```
./scripts
|-- 0_demography_SAs.R
|-- 1_deforestation_dataset_SA4.R
|-- 2_deforestation_model_SA4.R
|-- 3_deforestation_forecast_PS4.R
```

```
|-- data
|   |-- 0_data_demography_SAs.txt
|-- fragindex
|   |-- recl.txt
|   |-- r.forestfrag
|-- outputs
    |-- demography
    |-- forecast
    |-- model
```

Four scripts are available: `0_demography_SAs.R` is an R script to estimate the parameters of the demographic model using the function `demography()` of the `phcfM` R package, `1_deforestation_dataset_SA4.R` is an R/GRASS script to prepare the data-set for the deforestation model from the geographic data layers of the `phcfM_SM` GRASS location, `2_deforestation_model_SA4.R` is an R script to estimate the parameters of the deforestation model using the function `deforestation()` of the `phcfM` R package and `3_deforestation_forecast_PS4.R` is an R/GRASS script to forecast deforestation and carbon dioxide emissions for project site #4.

The `./scripts/data` folder includes the population census data file `0_data_demography_SAs.txt` used for the demographic model. Running the `1_deforestation_dataset_SA4.R` script generates the file `1_data_deforestation_SA4.txt` (the data-set used for the deforestation model) in the `./scripts/data` folder. The `./scripts/fragindex` folder includes the GRASS function `r.forestfrag` to compute the forest fragmentation index. The `./scripts/outputs` folder includes the outputs for the demographic model, the deforestation model and the deforestation forecast.

7 Appendix 7: Mathematical formulas for model performance indexes

Given a confusion matrix for comparing observations and model predictions for forested and deforested pixels (Table S1), several model performance indexes can be computed (Table S2) following definitions from Liu *et al.* (2011) and Pontius *et al.* (2008).

		Observations		Total
		0 (forest)	1 (non-forest)	
**Predictions**	0	n_{00}	n_{01}	n_{0+}
	1	n_{10}	n_{11}	n_{1+}
**Total**		n_{+0}	n_{+1}	n

Table S1: **Confusion matrix to assess model performance.**

Overall Accuracy	$\text{OA} = (n_{11} + n_{00})/n$
Figure Of Merit	$\text{FOM} = n_{11}/(n_{11} + n_{10} + n_{01})$
Sensitivity	$\text{Sen} = n_{11}/(n_{11} + n_{01})$
Specificity	$\text{Spe} = n_{00}/(n_{00} + n_{10})$
True Skill Statistic	$\text{TSS} = \text{Sensitivity} + \text{Specificity} - 1$
Cohen's Kappa (K)	$\text{K} = (\text{OA} - \alpha)/(1 - \alpha)$
	with $\alpha = (n_{1+}n_{+1} + n_{0+}n_{+0})/n^2$

Table S2: **Definitions of model performance indexes from the confusion matrix.**

8 Appendix 8: Comparison with Land Change Modeler and Dinamica EGO

8.1 Annual deforestation rates are biased when variable time-intervals are not taken into account

Last versions of Land Change Modeler (Selva) and Dinamica EGO (2.0) don't support the use of mosaics of satellite images with variable time-intervals between land-cover observations. They only accept a fixed time-interval between two dates to compute the annual deforestation rate. For Land Change Modeler, the fixed time-interval corresponds to a difference between two years and is thus constrained to be an integer (in yr). For Dinamica EGO, the fixed time-interval is not constrained to be an integer and the unit is defined by the user. For each study area and each time-period, we used the approach proposed by these two programs to compute the annual deforestation rate (θ). We used a weighted mean to estimate the average time interval between land cover observations (Y_{wm} in yr). The weights corresponded to the area (in km^2) of the scene with a given time interval. The computation of the weighted mean was not possible using LCM or Dinamica EGO. We had to use an external GIS software (GRASS) to perform the computation. We used Y_{wm} as the fixed time-interval for Dinamica EGO and the nearest integer Y_{int} as the fixed time-interval for LCM. We compared the results obtained with LCM and Dinamica EGO with our annual deforestation rate estimates which were obtained using the phcfM R package (Table S3).

All programs			LCM		D. EGO		phcfM
Study area	Period	θ'	Y_{int}	θ	Y_{wm}	θ	θ
SA1	1	9.159	11	0.87	10.97	0.87	0.87
	2	2.718	3	0.91	2.81	0.98	0.98
SA2	1	9.495	4	2.46	4.10	2.41	2.40
	2	11.560	5	2.43	4.95	2.45	2.45
SA3	1	5.597	4	1.43	4.45	1.29	1.28
	2	4.298	5	0.87	5.13	0.85	0.85
SA4	1	4.133	4	1.05	3.61	1.16	1.16
	2	6.520	6	1.12	5.56	1.20	1.20
SA5	1	2.788	6	0.47	5.56	0.51	0.51
	2	2.598	6	0.44	5.56	0.47	0.47

Table S3: **Comparison of annual deforestation rate estimates using LCM, Dinamica EGO and the phcfM R package.**

Using LCM fixed time-interval (constrained to be an integer), the annual deforestation rate was biased. The bias increased with the relative error regarding the time-interval. For example, for period 1 and study area 4, the weighted mean for time-interval was equal to 3.61 and the time-interval in LCM was thus fixed to 4 years, inducing an error of $100 \times ((4 - 3.61)/3.61) \simeq 11\%$ for the time-interval estimate and an underestimation of the estimated annual deforestation rate which decreases from 1.16 to 1.05 $\%.\text{yr}^{-1}$. Bias for annual deforestation rates might be rather small but errors accumulate with time. Error accumulation can potentially lead to significant differences regarding predictions of deforestation and carbon dioxide emissions.

When using a weighted mean for specifying the time-interval with Dinamica EGO, the annual deforestation rate was not biased and the results were the same as when using the phcfM R package. Nevertheless, as said previously, the weighted mean had to be computed outside Dinamica EGO with another GIS program such as GRASS. Moreover, for more complex models including factors (such as the population density) to explain the annual deforestation rate, the weighted mean cannot be used. An adapted statistical approach is necessary in this case, such as the one described through Equation 2 in the main text of the article. This statistical approach is made available in the phcfM R package through the use of the `deforestation()` function.

8.2 Deforestation and carbon dioxide emissions are largely underestimated when demographic expansion is not taken into account

LCM and Dinamica EGO do not allow estimating the population density effect on deforestation intensity and use a fixed annual deforestation rate to project deforestation in the future. Using study area 4 (moist forest near Fort-Dauphin) as an example, we projected the deforestation and the carbon dioxide emissions from 2010 to 2030 assuming a fixed annual deforestation rate of 1.20%.yr^{-1}. On the contrary, for our projections, the annual deforestation rate increased up to 1.42 %.yr^{-1} in 2030 in association with demographic expansion. In the following figure, projections assuming a fixed annual deforestation rates (in color) were compared with projections assuming an increasing annual deforestation rate (in grey).

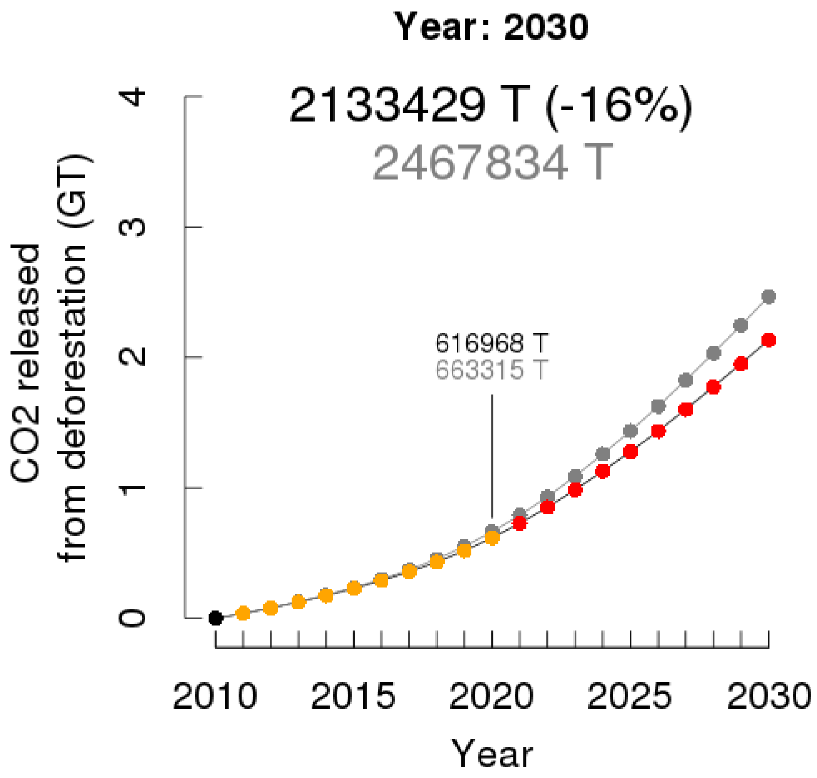

Figure S7: **Projections of carbon dioxide emissions when demographic expansion is not taken into account.**

When the demographic expansion is not taken into account, the carbone dioxide emissions are largely underestimated (-16% between 2010 and 2030 for study area 4). For study area 3 near Fandriana, which already has a high population density, the intensity

of deforestation is likely to increase dramatically from 2.94 to 4.11%.yr^{-1} between 2010 and 2030 due to the exponential population growth. In this case, projections that do not account for demogaphic expansion underestimate carbon dioxide emissions of -20% (result not shown).

References

Chib S, Carlin BP (1999) On MCMC sampling in hierarchical longitudinal models. *Statistics and Computing*, **9**, 17–26.

Liu C, White M, Newell G (2011) Measuring and comparing the accuracy of species distribution models with presence-absence data. *Ecography*, **34**, 232–243.

Martin AD, Quinn KM, Park JH (2011) MCMCpack: Markov chain Monte-Carlo in R. *Journal of Statistical Software*, **42**, 22.

Pemstein D, Quinn KM, Martin AD (2011) The Scythe statistical library: an open source C++ library for statistical computation. *Journal of Statistical Software*, **42**, 1–26.

Pontius R, Boersma W, Castella JC, *et al.* (2008) Comparing the input, output, and validation maps for several models of land change. *The Annals of Regional Science*, **42**, 11–37.
